# Comparison of Quantitative light-induced fluorescence-digital (QLF-D) images and images of disclosed plaque for planimetric quantification of dental plaque in multibracket appliance patients

**DOI:** 10.1038/s41598-020-61454-9

**Published:** 2020-03-11

**Authors:** Katharina Klaus, Tabea Glanz, Alexander Georg Glanz, Carolina Ganss, Sabine Ruf

**Affiliations:** 1 Department of Orthodontics, Justus-Liebig-University Gießen, Germany (Schlangenzahl 14, 35392 Gießen, Germany; 2 Private Practice, Lüdenscheid, Germany (Dr. Arndt Himmen, Freiherr-vom-Stein-Str. 24, 58511 Lüdenscheid, Germany; 3 Private Orthodontic Practice, Zweibrücken, Germany (Dr. Michael Wagner, Poststr. 5, 66482 Zweibrücken, Germany; 4 Department of Conservative and Preventive Dentistry, Justus-Liebig-University Gießen, Germany (Schlangenzahl 14, 35392 Gießen, Germany

**Keywords:** Paediatric research, Risk factors

## Abstract

The purpose of the present cross-sectional clinical study was to check the ability of plaque detection and quantification by QLF-D against conventional digital photographs of disclosed plaque in multibracket appliance (MB) patients. 20 patients were included according to the following criteria: (1) upper and lower jaw treated by MB appliance, (2) patients being 16 years of age or older, (3) all central and lateral incisors as well as canines *in situ*, (4) absence of developmental defects, carious lesions, surface fillings, prosthetic restorations or recessions greater than 1/3 of root length in central/lateral incisors and canines as well as (5) declaration of consent. QLF-D and conventional photographs were analyzed planimetrically regarding plaque coverage on buccal and oral surfaces of central/lateral incisors and canines. The conventional photographs of stained plaque served as gold standard. On average, in QLF-D pictures 20.7% ± 17.4 of the tooth surfaces were covered with plaque, while the conventional photographs of disclosed plaque presented a mean plaque-covered area of 36.2% ± 23.5. The Bland-Altman plot for both imaging modalities showed a very large inconsistent scattering with both negative and positive deviations. The method discrepancy increased with increasing plaque coverage, thus indicating a systematic method error. On average, the deviation of the methods from the optimal line of accordance was −15.5%. In patients wearing MB appliances, there was no clinical significant agreement regarding the plaque-covered tooth surface depicted by QLF-D respectively conventional images of disclosed plaque. Due to the large method discrepancy, QLF-D is currently not reliable for precise plaque quantification in MB patients.

## Introduction

Plaque control during multibracket appliance (MB) treatment is a special challenge because a MB inevitably increases the number of retentive niches for plaque accumulation. Detection and quantification of dental plaque plays an important role in everyday practice, both for patients’ education and motivation. In addition, it is also important for clinical research.

Due to the buccal and/or lingual attachments and wires of a MB appliance, modified plaque indices have been developed for use in orthodontic patients^1,2^. Although plaque indices allow for a fast assessment of the amount and localization of plaque, they have their disadvantages especially for research purposes, as due to their subjective nature time- and cost-intensive examiner training for calibration and reliability is required. Furthermore, the comparison of different study results is hindered by the variety of indices used. In addition, due to their ordinal scaled nature, the discriminating capacity of some indices is insufficient because of their limited number (commonly four^2–4^) of categories. Thus, for the assessment of small plaque differences and the enhancement of the statistical power in clinical trials, interval scaled measurements would be beneficial^5^.

Planimetric methods have been described to overcome the above mentioned disadvantages^6–9^. After disclosing the plaque, photographs of the tooth surfaces are taken and the amount of plaque is calculated as a percentage of tooth surface coverage^6–9^. However, plaque disclosure remains necessary and requires a subsequent time-consuming professional tooth cleaning.

Quantitative light-induced fluorescence (QLF) could possibly provide an alternative solution for plaque assessment. QLF has been previously established for caries detection, caries monitoring and effects of oral hygiene and remineralization approaches^10–13^, but could also be useful for plaque quantification^3,14^ and has already shown its capacity and validity compared to oral hygiene indices^15–17^. Compared to disclosed plaque, contradictory results concerning its validity have been reported^10,18,19^. QLF is based on the fact, that plaque shows fluorescence in green, orange and red, if stimulated with light of specific wavelengths^20^. The intensity of red fluorescence is due to synthesized endogenous porphyrins of oral bacteria and has been shown to correlate with age and thickness of the biofilm^21,22^.

QLF was further developed into Quantitative light-induced fluorescence-digital (QLF-D Billuminator, Inspector Research System, Amsterdam, The Netherlands) which enhances the degree of red fluorescence. The system consists of an illumination tube with eight violet-blue light-emitting diodes (LEDs; 405 ± 20 nm) and four white LEDs (broad spectrum, 6500 K) on a ring tube around a 60-mm macro lens and a modified filter set. Excited by ultraviolet light, the red fluorescence is captured on a high-resolution image for further processing. Using either the blue or the white LEDs, the camera system is able to produce QLF-D images as well as conventional digital photographs.

Most QLF-D studies on plaque detection have been performed *in vitro*^21–26^. From the current *in vivo* studies^15,16,18,27–29^, only one^27^ had a sample of MB patients. Nevertheless, the purpose of the latter study was not primarily the detection and quantification of plaque, but the use of QLF-D images as a visual aid for oral hygiene reinforcement^27^.

Therefore, the purpose of the present cross-sectional *in vivo* study in MB patients was to compare plaque detection and planimetric quantification by (1) QLF-D with (2) conventional digital photographs of disclosed plaque. The null hypothesis was to detect no difference between the plaque scores derived by both methods.

## Subjects and Methods

### Subjects

The study was performed according to the Declaration of Helsinki and the guidelines of Good Clinical Practice. Ethical approval was granted by the ethical committee of the medical faculty at the Justus-Liebig-University Gießen, Germany (No. 58/13, date of approval: 28.05.2013).

Participants were recruited on a voluntary basis in the Department of Orthodontics at the Justus-Liebig-University Gießen, Germany. Interested patients received oral and written information about the study and had a minimum time of 24 hours to consider participation.

20 patients (6 male, 14 female, mean age 18.6 ± 4.7 years, age range 16–31 years) were selected according to the following inclusion criteria:

- MB appliance in both upper and lower jaw
- age ≥ 16 years
- presence of all canines, lateral and central incisors in the upper and lower jaw (teeth 13–23, 33–43 according to the FDI scheme (Fédération Dentaire Internationale))
- neither developmental defects, carious lesions, fillings on the labial surfaces, crowns or recessions > 1/3 of root length in the canines, lateral and central incisors (teeth 13–23, 33–43)
- written informed consent signed by patients and parents.

Exclusion criteria were:

- serious general diseases with possible systemic, medicational and/or manual effect on plaque formation and accumulation
- gingival or periodontal inflammation
- smoking habits
- antibiotic drug intake.

In order to ensure that every patient presented at least a certain amount of plaque to be quantified, all patients were advised to refrain from oral hygiene measures the day of the study appointment^15,18,19,28^.

### Photo status

**Figure 1:**
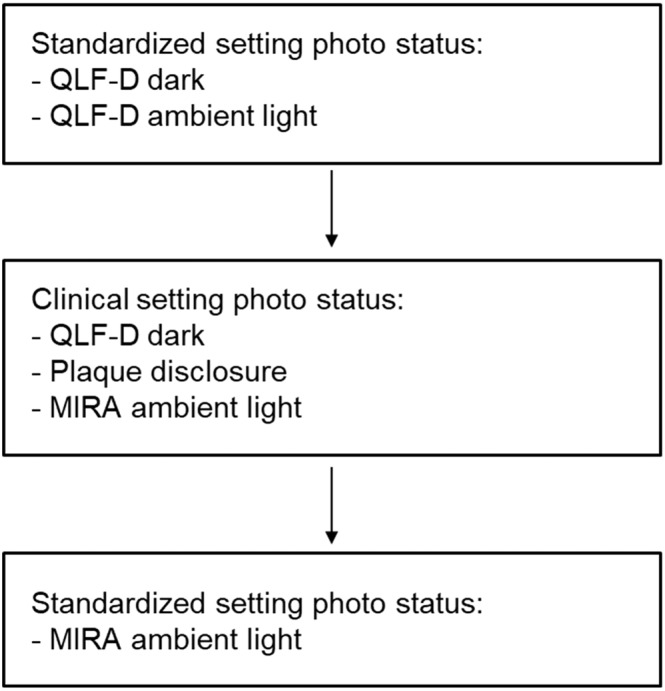
Flowchart of photo sequence for conventional (stained plaque) and QLF-D images.

**Figure 2:**
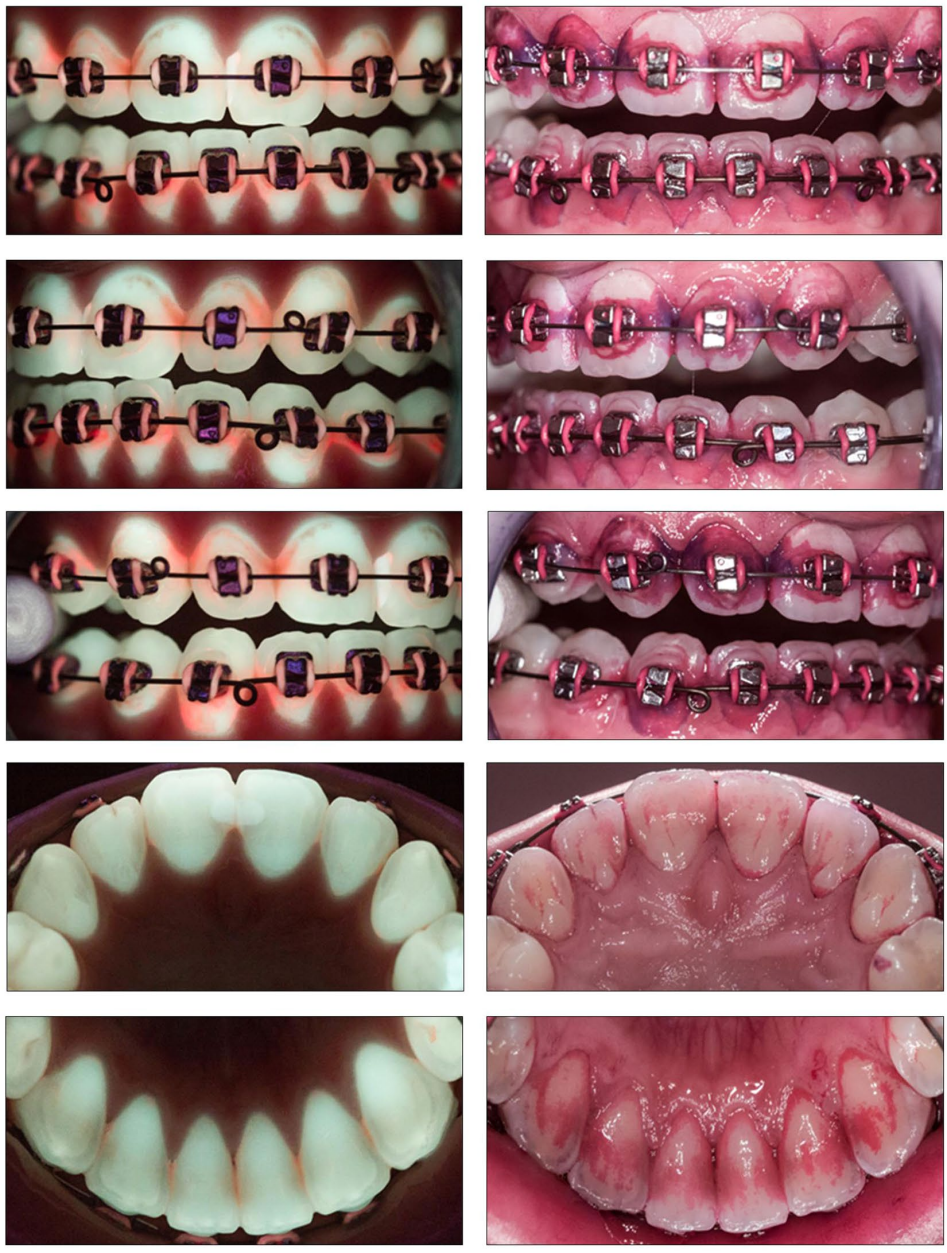
Complete photo status of QLF-D and conventional (stained plaque) pictures of the same patient.

Both QLF-D and conventional digital photographs were taken under standardized and clinical conditions (see flowchart Fig. 1) with the same camera system (QLF-D Billuminator, Inspector Research System, Amsterdam, The Netherlands). The different camera adjustments for the QLF-D as well as the conventional digital photographs are presented in Table 1. Two cotton rolls were placed between the upper and lower jaw in the molar region to avoid incisor overlap upon closing. A lip retractor was used for full display of all incisors and canines. Three labial pictures per patient were taken: frontal view for the upper and lower incisors and left/right lateral views for the canines. Lingual pictures of the incisors and canines were taken using a mirror (Dent-o-care photo mirror, Dent-o-care, Höhenkirchen, Germany) (Fig. 2).

**Table 1:** Camera adjustments (Canon EOS 550d) for conventional (stained plaque) and QLF-D images.

	conventional (Mira)	QLF-D
Shutter Speed	1/30 s	1/30 s
Aperture	f8.0	f5.6
ISO Sensitivity	ISO 1600	ISO 1600
White balance	Manual	Daylight
- Temp	2000	5200
- Tint	−8	+3
Illumination	normal LED	blue LED

For the standardized setting, photographs were taken in a room without windows and thus constant ambient light conditions. Patients sat in front of the camera stand with their chin placed in a headrest. Headrest and camera tubus had a fixed distance of 5 cm and the tubus was adjusted rectangular to the teeth. One set of QLF-D images each was taken (1) under dark conditions and (2) with ambient light. In addition, the conventional images were taken under ambient light.

For the clinical setting, patients sat in a dental chair, leaning their head against the headrest of the dental unit. One investigator held the camera manually with a distance as close as possible to 5 cm and as rectangular as possible to the teeth. QLF-D pictures were taken under dark conditions with shutters closed, the conventional images were taken under ambient light.

First, all QLF-D images were taken, subsequently the dental plaque was disclosed by means of Mira-2-Ton solution (Hager & Werken, Duisburg, Germany). The disclosing agent was applied with a saturated foam pellet, thereafter the patients rinsed with water for 10 s, and the conventional images at both settings were taken. Finally, the teeth were cleaned professionally. The rinsing time for the disclosing agent was set at 10 s, because no validated time exists in literature and even the manufacturer gives no clear instructions. Comparable studies^18,19^ give either no information on rinsing time^18^ or describe they allowed their patients “to rinse with tap water once”^19^, which should be within the scope of 10 s.

### Plaque measurement

**Figure 3:**
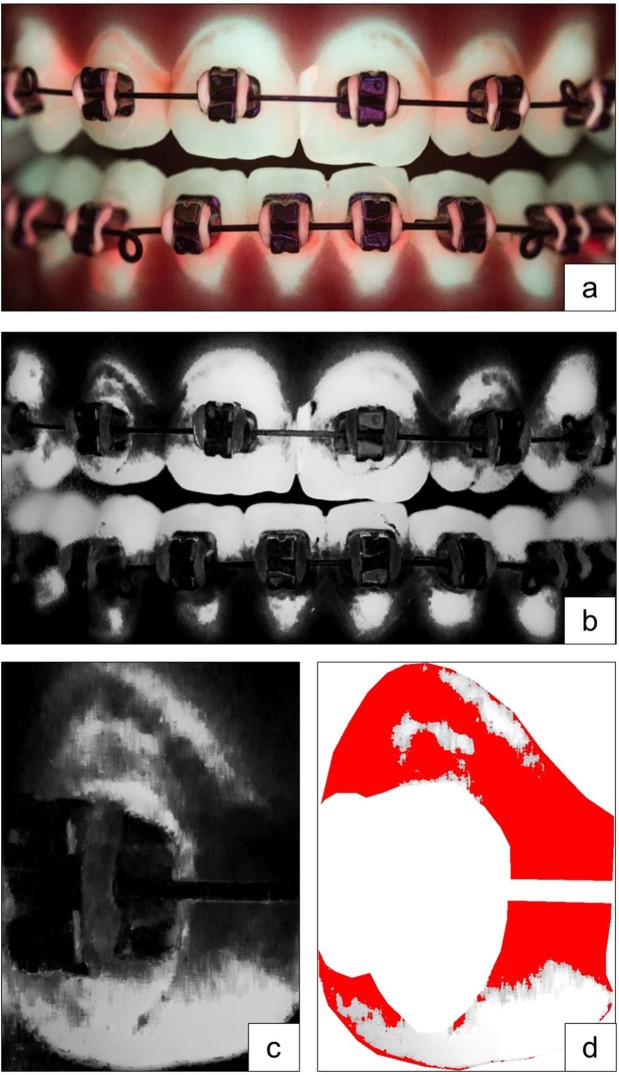
Identification of the “region of interest” for plaque measurement, (**a**) raw data file, (**b**) converted into gray-scale, (**c**) identification of the “region of interest”, example shows tooth 12, (**d**) visualization of all pixels representing plaque covered areas according to the brightness thresholds.

The filenames of all pictures were pseudonymised thus blinding the evaluator for the individual patient, setting and lightning condition. The raw data files were imported into Adobe Photoshop Lightroom 5.7.1 (Adobe Systems, San José, CA, USA) and converted into gray-scale. In several consensus rounds of the authors, a brightness threshold for conventional and QLF-D images was determined for defining if a pixel was covered with plaque or not. Considering an 8-bit gray scale, a value of 0 represents pure black and a value of 255 pure white. For the conventional images of disclosed plaque, the threshold was set at 90, while for QLF-D, threshold was set at 180. Plaque was measured with an ImageJ 1.47q-Macro (Wayne Rasband, National Institute of Mental Health, Bethesda, MD, USA). First, the “region of interest” was defined for every picture by manually masking the entire tooth surface and cutting out brackets and wires. Then, the plaque-covered areas were measured by batch-processing, using the above mentioned brightness thresholds (Fig. 3). Plaque coverage was measured in percentage of the total bracket- and wire-free tooth crown surface. Measurements were performed separately for labial and lingual surfaces, incisors and canines and for the upper and lower jaw.

### Inter- and intraexaminer reliability

The examiners were extensively trained and calibrated before starting the investigation. The reproducibility of the planimetric evaluation was tested in a previous study, in which the examiners were actively involved^29^. For testing the reproducibility of the planimetric analysis, a complete set of images was analyzed by two investigators twice. The mean difference of results achieved by the two investigators (inter-examiner reliability) was 0.82 ± 5.4% surface plaque coverage for conventional pictures of disclosed plaque and 1.1 ± 3.2% for QLF-D. The mean difference of surface plaque coverage for the repeated analysis (intra-examiner reproducibility) was 1.8 ± 5.6 for conventional pictures and 2.1 ± 4.3 for QLF-D. The correlation coefficients for all procedures ranged between 0.97 and 0.99, which indicates an excellent agreement.

### Statistical methods

Statistics were performed by a professional statistician using IBM SPSS Statistics, Version 23 (IBM Corporation, Armonk, NY, USA). Data did not show a normal distribution (Kolmogorov-Smirnov-test, Shapiro-Wilk-test), thus all further comparisons between methods, settings, jaws and tooth surfaces were performed by means of non-parametric tests (Wilcoxon test for method comparison, Friedman test for setting, jaw and surface differences). Despite the non-normative distribution of data, no skewed distribution pattern was detectable. Therefore, the mean values were used for further group comparisons. Agreement between the two methods was analyzed by Bland-Altman-Plots. The significance level was set at 0.05. Because no sample size calculation was undertaken a priori, a post-hoc power analysis was performed using G*power, Version 3.1 (HHU Düsseldorf, Düsseldorf, Germany).

## Results

**Figure 4:**
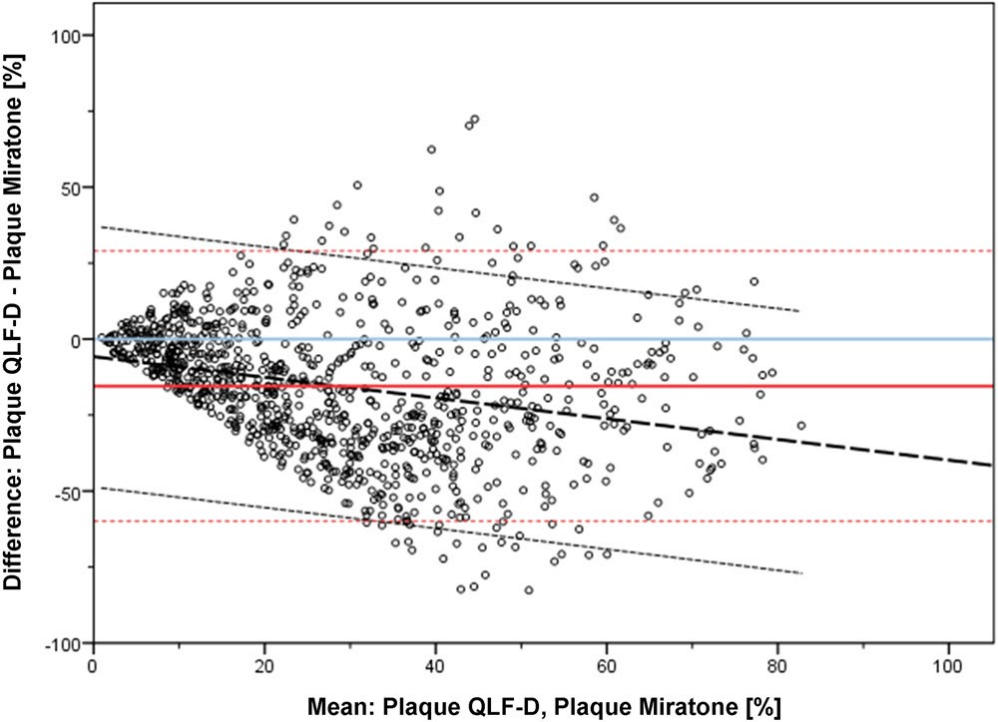
Bland-Altman plot for QLF-D and conventional (disclosed plaque) imaging irrespective of jaws, tooth type, tooth surface, setting and lighting conditions. Bold red line: Mean difference (%) of both methods, blue line: optimum line of accordance, dotted red lines: 95% limits of agreement, bold dotted black line: regression line, dotted black lines: confidence limits of regression line.

The mean plaque coverage of all analyzed surfaces and all patients was 20.7% ± 17.4 for QLF-D pictures and 36.2% ± 23.5 for Miratone stained conventional photographs (Table 2). The Bland-Altman plot for both imaging modalities showed a very large inconsistent scattering with both negative and positive deviations. The method discrepancy increased with increasing plaque coverage, thus indicating a systematic method error (Table 3, Fig. 4). QLF-D and Miratone images presented a statistically significant difference of the planimetrically assessed plaque coverage (p < 0.001). On average, the deviation of the methods from the optimal line of accordance was −15.5%. In other words, QLF-D showed 15.5% less absolute plaque covered tooth surface than did Miratone staining. Therefore, the null hypothesis had to be rejected.

**Table 2:** Descriptive statistics of plaque coverage percentage of QLF-D and conventional imaging.

Parameter	QLF-D	Mira
Mean	20.7	36.2
Standard deviation	17.4	23.5
Minimum	0.1	0.6
Maximum	86.7	98.1

**Table 3:** Descriptive statistics of the Bland-Altman-analysis of QLF-D and conventional imaging. Asterisks indicate a highly significant result (p < 0.001).

Parameter	Difference QLF-D/Mira (%)
Mean	−15.5***
Standard deviation	22.6
Minimum	−82.6
Maximum	72.4

A statistical analysis of the different settings (standardized/clinical), jaws (upper/lower), teeth (incisors/canines) and surfaces (oral/vestibular) was additionally undertaken (Supplementary material: Figure S1, Table S1). However, keeping in mind the large systematic method error, we refrained from a detailed description of the results. In summary, these analyses showed that the systematic method error was basically independent of the settings, jaws, tooth types and tooth surfaces analyzed. Method differences were however more dependent on the oral region than on the setting/light conditions.

The post-hoc power analysis revealed that the correlation of the Bland-Altman-analysis (r = 0.432), given an alpha-error of 5%, had a power of 66.1% and thus the study was slightly underpowered.

## Discussion

The insertion of a MB appliance is not only a challenge for the oral hygiene of patients due to the iatrogenic plaque retentive niches, it also induces a shift in the number and composition of oral microbiota^30–34^. Oral microbiota counts peak at about three months after insertion of a fixed appliance and show a slight decrease thereafter^32–34^, nevertheless MB patients present consistently higher counts of oral microbiota compared to their pretreatment situation^32–34^. Therefore, proper plaque control during orthodontic treatment is vital to prevent plaque-dependent side effects of MB treatment in the form of initial carious lesions or gingival/periodontal inflammation.

Initially, the QLF technique was developed and proved to be a suitable tool for diagnosis and monitoring of initial carious lesions^10–13,35,36^. As a side effect, red autofluorescence of plaque was observed^37^. Until now, this phenomenon is still under investigation: Protoporphyrin IX is suggested to emit red fluorescence in dental plaque^38,39^. This fluorescence is associated with the etiological changes during plaque maturation rather than with the characteristics of single microbial species^14,21,25^. Increased thickness, age, maturation and cariogenicity of biofilms were found to be associated with higher intensities of red fluorescence *in vitro*^22,24,26^, while *in vivo*, on the other hand, no correlation with the cariogenicity could be found^40^. Thus, the above mentioned change in number and composition of oral microbiota during MB treatment could have influenced the degree of fluorescence. One research group^23^ investigated red fluorescence of oral biofilms *in vitro* and found it to be observed during early biofilm formation and to be linearly related to the total mass of biofilm. They suspected red fluorescence to be more closely associated with the level of gingival inflammation rather than caries^23^. Considering the current literature, it has to be concluded, that the phenomenon of red autofluorescence of dental plaque as captured by the QLF-D method is still lacking a solid research background.

Due to the fact that red fluorescence is related to the maturity of oral biofilms, a two tone disclosing agent was used in the present study (Mira-2-Ton solution, Hager & Werken, Duisburg, Germany). It contains aqua, sodium benzoate, potassioum sorbate, acid red 92 (CI 45410) and acid blue 9 (CI 42090). According to the manufacturer it differentiates between “new” plaque (stained pink) and “old” plaque (stained blue). Even though two tone disclosing solutions are widely used in dentistry and represent the gold standard in plaque disclosure, the underlying principles of plaque staining are not yet fully understood. Whereas the pink dye is supposed to adhere to all plaque that is present and perhaps even to unbound proteins in the oral cavity^15,19,41^, the blue staining is a result of diffusion into thicker plaque areas. Nevertheless, the adherence of the blue dye is weaker than the pink dye. Continuously *in vitro* washing can dissolve the blue dye completely, in contrast to the pink dye^41^.

The present patient sample was advised to refrain from oral hygiene for the day of study appointment, which represents a time span ranging in the middle of comparable studies^15,18,19,28^. Given a perfect plaque removal by the patient the evening before, this should have resulted in young plaque (pink staining) at the study appointment. Because there was no professional tooth cleaning at baseline the study results reflect the individual oral hygiene levels of the patients plus plaque accumulation since the evening before. This approach seemed acceptable as the study did not aim at the absolute level of plaque accumulation but the differences between the quantification methods.

The first researchers^14^ who compared the amount of plaque identified by red fluorescence and disclosed plaque (one tone fluorescing agent) in non-MB patients reported a correlation between both methods, despite the fact that plaque coverage of disclosed plaque was approximately 62% larger. Furthermore, using the disclosing agent, plaque was identified despite the absence of red fluorescence, which underlines the higher sensitivity of the disclosing agent. In the present study, QLF-D underestimated the amount of dental plaque by on average 15.5% in comparison to the plaque disclosing agent. However, there were large deviations in QLF-D detected plaque in both directions. The method discrepancy increased with increasing plaque coverage. As figure S1 showed, variations in photographic settings did not influence the method discrepancy between QLF-D and disclosed plaque substantially. In a similar study in non-MB patients, plaque formation over 72 hours was monitored by both QLF-D and conventional pictures of disclosed plaque^29^. As in the present study, the method discrepancy increased with increasing plaque coverage, indicating a systematic method error (5.4 to 11.1% underestimation by QLF-D in comparison to the plaque disclosing agent). With increasing time for plaque formation, also the method error increased^29^.

A significant similarity between QLF-D detected plaque areas and disclosing agent stained areas of older plaque (blue) is reported^18^. Correspondingly, a significant difference with respect to red stained areas of younger plaque, which were approximately three times greater than the blue/QLF-D areas, was shown. Interestingly, the scatterplot comparing the conventionally stained old plaque (blue) areas with the QLF-D areas showed a comparable systematic method discrepancy with increasing amounts of plaque as in the present study.

In contrast to the above mentioned results^18^, another research group^19^ found a weak to moderate correlation between QLF-D and the old (blue) plaque areas, whereas the comparison between QLF-D and the combined old and new (pink and blue) areas revealed a moderate to strong correlation. Alike the results in the present study, plaque areas on the QLF-pictures were on average lower than the areas measured for disclosed plaque, underlining a potential underestimation of plaque coverage by QLF-D compared to plaque disclosure, which is also confirmed by the results of several other studies^5,14,18^.

To overcome the limitations of staining, an attempt to validate QLF-D plaque assessment against two plaque indices was made^15^, one of them mainly evaluating plaque thickness. Furthermore, the intensity of plaque fluorescence by QLF-D by using the red/green ratio (ΔR) was calculated, and the highest (but even moderate) correlation existed between the thickness-dependent plaque index and ΔR30, meaning that “the redness difference between the tooth and the plaque is at least 30%”^15^. The percentage plaque area for every ΔR was also calculated, but unfortunately not published. In contrast, another study of the same research group^18^ found the correlation between QLF-D and disclosed plaque to be independent of ΔR in the same study population^15,18^. Given these contradictory results for ΔR in literature, the fact that ΔR was not considered in the present study should not represent a major limitation of the study.

### Limitations of the study

Possible limitations of the current study could be seen in the method of manually masking the tooth surfaces and cutting out brackets and wires in both QLF-D and Miratone stained pictures. As disclosing agents stain a wider area of plaque as QLF-D, the effect of removing the brackets could have had a higher impact on the QLF-D images, thus contributing to the reduced plaque scores. To minimize this limitation, intra- and interexaminer reliability was tested in a previous study and found to be excellent^29^. Additionally, many studies dealing with QLF for the quantification of white spot lesions show a high repeatability and reproducibility in identifying the decalcified areas for trained investigators^35,36^. Furthermore, plaque coverage in MB patients also affects the surfaces of brackets and ligatures and even the color of ligatures influences plaque disclosure in this area^42^. Therefore, and to enhance the comparability with literature, the areas of brackets and wires were cut out.

For recognition of plaque-covered pixels in QLF-D and Miratone stained photographs, brightness thresholds of 180 for QLF-D and 90 for Miratone stained images were set by consensus rounds of the authors of the present study and are thus, to a certain extent subjectively. However, a certain level of subjectivity seems to be inevitable for trials using software-aided planimetric measurements for methodological reasons^17^.

No sample size calculation was conducted for the present study, because of its non-interventional design. Sample sizes in comparable studies of QLF-D plaque assessment in non-MB patients range from one to 51 patients^5,14,15,18,19^, while studies of planimetric plaque assessment independently of the technique used in MB patients comprise between 20 and 52 patients^27,42–44^. Thus, the present sample size of 20 patients was average related to comparable QLF-D studies in non-MB patients^5,14,16,18,19^ and small related to plaque assessment studies in MB-patients^27,42–44^. The post-hoc power analysis revealed a power of 66.1%, which is 14% less than the widely used standard of 80% and could have led to a slightly higher beta-error. To achieve a power of 80%, a total sample size of 29 patients would have been necessary. However, the level is still acceptable for an explorative study, especially given the large methodological differences detected. Further studies should aim to establish a larger patient sample in order to reduce the beta-error as much as possible.

Investigating the use of QLF-D and non-stained conventional digital photographs for oral hygiene remotivation in MB patients by using the pictures as visual aids in oral hygiene reinforcement showed a plaque reduction over a 6 month period for both groups^27^. Additionally, all patients with QLF-D pictures were in favor of monitoring oral hygiene in this way throughout the entire treatment, whereas only 81% of patients with conventional pictures suggested that. In the present study, the use of QLF-D for motivational purposes was not investigated. Given the fact that QLF-D could be time saving in clinical routine, because plaque disclosure with subsequent need for professional tooth cleaning is not necessary, it would be desirable to use QLF-D in patient motivation. Keeping the large systematic method error in mind, QLF-D currently does not seem to quantify the extent of plaque precise enough for both a clinically and for experimentally reliable plaque quantification. Nevertheless, QLF-D could be a helpful tool to demonstrate the location of plaque to patients and could thus to a certain extent be beneficial for oral hygiene education^27^.

All in all, the authors expected a stronger correlation between QLF-D and disclosed plaque without the large deviations detected in the study. The available literature shows that QLF-D has a good validity and reliability for plaque quantification compared to plaque indices^15–17^, despite reported differences in plaque quantification between QLF-D and disclosed plaque^14,18,19^. Although the Bland-Altman-analysis of the present study was slightly underpowered, in any case the large interindividual deviations and the higher scattering with increasing amounts of plaque is unlikely to have been influenced by a higher power respectively a larger patient sample.

Taking into account that the two methods compared are based on various underlying principles which are not yet fully understood, the presented differences in plaque detection could also reflect the different sensitivity of the methods themselves. A recent study evaluated the red fluorescence of subjects with experimental gingivitis and identified groups of subjects with low, moderate and high fluorescence levels^45^. Given the fact that microorganisms emit different fluorescence in monocultures than in biofilms and the degree of red fluorescence also depends on specific nutrients in the culture media^21^, the amount of fluorescence could also be influenced on a personal subject level due to individual microbial composition, nutrition habits or active ingredients of oral hygiene products. If QLF-D would be that sensitive, the disclosing agent mainly basing on diffusion principles is incapable to present comparable results to QLF-D which could be detected by planimetrically plaque measurement.

## Conclusion

Dental plaque quantification in MB patients by QLF-D in comparison to Miratone stained digital photographs underestimated plaque coverage on average by 15.5%, with very large deviations in both directions. The method discrepancy increased with increasing plaque coverage. Due to the fact that the complete underlying principles for QLF-D as well as Miratone plaque disclosure are not yet fully understood, further research is needed to assess whether QLF-D or Miratone solution might serve as a reliable tool for exact plaque quantification in future.

### Ethical approval

All procedures performed in this study involving human participants were in accordance with the ethical standards of the ethical committee of the medical faculty of Justus-Liebig-University Gießen, Germany (no. 58/13, date of approval: 28.05.2013) and with the 1964 Helsinki declaration and its later amendments.

### Informed consent

Informed consent was obtained from all individual patients included in the study, and, as many of them were younger than 18 years, the written consent form was signed by both parents.

## Supplementary information

Supplementary Dataset 1.

## Data Availability

The datasets generated during and/or analyzed during the current study are available from the corresponding author on reasonable request.
