# Supplementary material for: Comparison of Quantitative light-induced fluorescence-digital (QLF-D) images and images of disclosed plaque for planimetric quantification of dental plaque in multibracket appliance patients
Source: Sci Rep. 2020 Mar 11;10:4478. doi: 10.1038/s41598-020-61454-9 (PMC7066241; doi:10.1038/s41598-020-61454-9)
Supplement: Supplementary file 1 — Supplementary Dataset 1. [file 41598_2020_61454_MOESM1_ESM.pdf]

Comparison of Quantitative light-induced fluorescence-digital (QLF-D) images and images of disclosed plaque for planimetric quantification of dental plaque in multibracket appliance patients – Supplementary data

Dr. Katharina Klaus*[1], Tabea Glanz[2], Dr. Alexander Georg Glanz[3], Prof. Dr. Carolina Ganss[4], Prof. Dr. Sabine Ruf[1]

1 Department of Orthodontics, Justus-Liebig-University Giessen, Germany (Schlangenzahl 14, 35392 Giessen)

2 Private Practice, Lüdenscheid, Germany (Dr. Arndt Himmen, Freiherr-vom-Stein-Str. 24, 58511 Lüdenscheid)

3 Private Orthodontic Practice, Zweibrücken, Germany (Dr. Michael Wagner, Poststr. 5, 66482 Zweibrücken)

4 Department of Conservative and Preventive Dentistry, Justus-Liebig-University Giessen, Germany (Schlangenzahl 14, 35392 Giessen)

*Correspondence to:

Katharina Klaus

Department of Orthodontics, Justus-Liebig-University Giessen

Schlangenzahl 14

35392 Giessen

Germany

E-Mail: katharina.klaus@dentist.med.uni-giessen.de

Phone: +49 641 99 46 130

Fax: +49 641 99 46 119

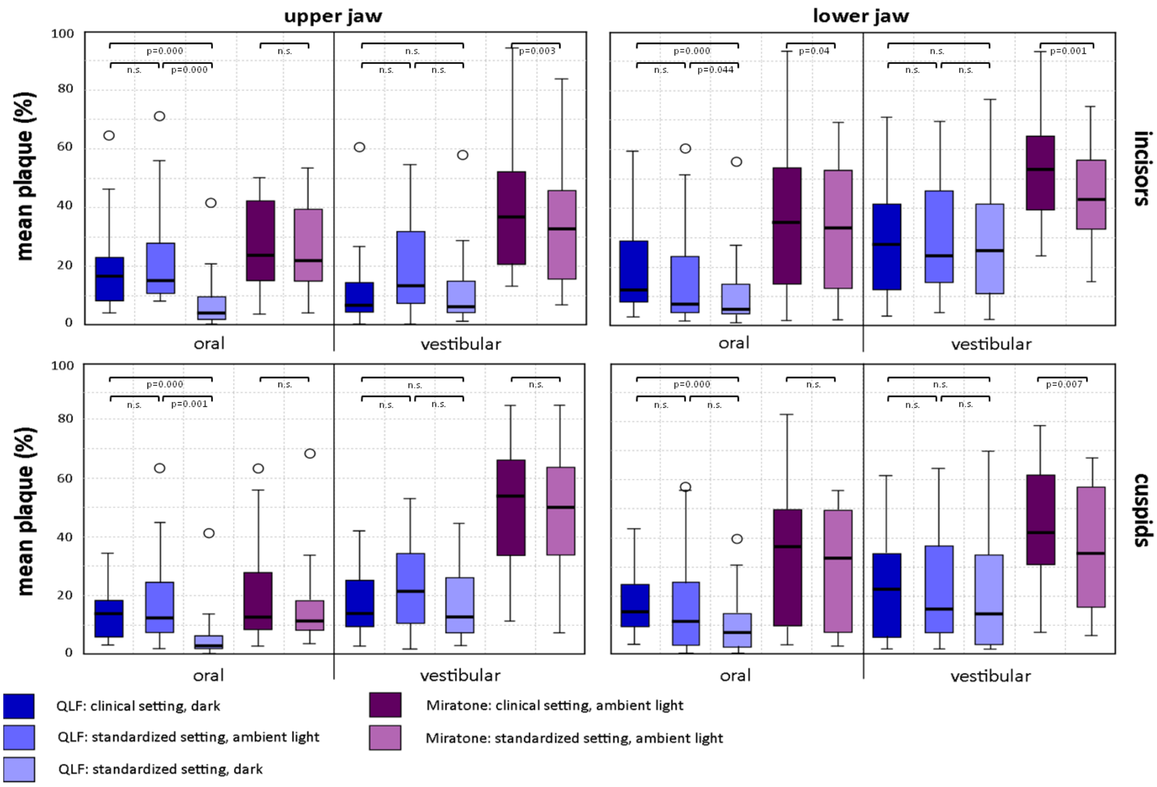

Supplementary figure S1:

Fig. S1 Mean plaque coverage (%) of QLF-D and conventional (disclosed plaque) images for different settings, jaws, tooth types and tooth surfaces; p-values are given for different settings, n.s. = not significant

Settings QLF-D

jaw	teeth	surface	MEAN stand. amb.	MEAN clin.	MEAN stand. amb. - MEAN clin.	p-value
upper	incisors	oral	23.69	19.43	4.26	n.s.
		vestibular	18.79	14.44	4.35	n.s.
	canines	oral	19.24	16.08	3.16	n.s.
		vestibular	24.39	18.52	5.87	n.s.
lower	incisors	oral	16.20	18.32	-2.12	n.s.
		vestibular	28.68	28.99	-0.31	n.s.
	canines	oral	17.98	18.90	-0.92	n.s.
		vestibular	24.17	23.03	1.14	n.s.
**jaw**	**teeth**	**surface**	**MEAN stand. dark**	**MEAN clin.**	**MEAN stand. dark - MEAN clin.**	**p-value**
upper	incisors	oral	8.28	19.43	-11.15	0.000
		vestibular	14.06	14.44	-0.38	n.s.
	canines	oral	7.52	16.08	-8.56	0.000
		vestibular	18.75	18.52	0.22	n.s.
lower	incisors	oral	10.95	18.32	-7.36	0.000
		vestibular	27.43	28.99	-1.56	n.s.
	canines	oral	11.17	18.90	-7.73	0.000
		vestibular	21.46	23.03	-1.57	n.s.
**jaw**	**teeth**	**surface**	**MEAN stand. dark**	**MEAN stand. amb.**	**MEAN stand. dark - MEAN stand. amb.**	**p-value**
upper	incisors	oral	8.28	23.69	-15.41	0.000
		vestibular	14.06	18.79	-4.73	n.s.
	canines	oral	7.52	19.24	-11.72	0.001
		vestibular	18.75	24.39	-5.65	n.s.
lower	incisors	oral	10.95	16.20	-5.25	0.044
		vestibular	27.43	28.68	-1.25	n.s.
	canines	oral	11.17	17.98	-6.81	n.s.
		vestibular	21.46	24.17	-2.71	n.s.

Settings Mira

jaw	teeth	surface	MEAN stand. amb.	MEAN clin.	MEAN stand. amb. - MEAN clin.	p-value
upper	incisors	oral	25.73	27.33	-1.60	n.s.
		vestibular	35.31	40.08	-4.76	0.003
	canines	oral	16.64	19.22	-2.58	n.s.
		vestibular	49.17	52.52	-3.35	0.021
lower	incisors	oral	32.02	36.09	-4.07	0.040
		vestibular	43.26	52.34	-9.08	0.001
	canines	oral	31.03	35.37	-4.34	n.s.
		vestibular	38.75	44.32	-5.57	0.007

Jaws QLF-D

teeth	setting	surface	MEAN lower jaw	MEAN upper jaw	MEAN lower jaw - MEAN upper jaw	p-value
incisors	clin.	oral	18.32	19.43	-1.11	n.s.
		vestibular	28.99	14.44	14.54	0
	stand. amb.	oral	16.20	23.69	-7.49	n.s.
		vestibular	28.68	18.79	9.89	0.021
	stand. dark	oral	10.95	8.28	2.68	n.s.
		vestibular	27.43	14.06	13.36	0.001
canines	clin.	oral	18.90	16.08	2.82	n.s.
		vestibular	23.03	18.52	4.51	n.s.
	stand. amb.	oral	17.98	19.24	-1.26	n.s.
		vestibular	24.17	24.39	-0.22	n.s.
	stand. dark	oral	11.17	7.52	3.65	n.s.
		vestibular	21.46	18.75	2.71	n.s.

Jaws Mira

teeth	setting	surface	MEAN lower jaw	MEAN upper jaw	MEAN lower jaw - MEAN upper jaw	p-value
incisors	clin.	oral	36.09	27.33	8.76	n.s.
		vestibular	52.34	40.08	12.26	0.002
	stand. amb.	oral	32.02	25.73	6.29	n.s.
		vestibular	43.26	35.31	7.94	0.006
canines	clin.	oral	35.37	19.22	16.15	0.017
		vestibular	44.32	52.52	-8.20	n.s.
	stand. amb.	oral	31.03	16.64	14.39	0.01
		vestibular	38.75	49.17	-10.42	0.011

Teeth QLF-D						
jaws	setting	surface	MEAN canines	MEAN incisors	MEAN canines - MEAN incisors	p-value
upper	clin.	oral	16.08	19.43	-3.35	n.s.
		vestibular	18.52	14.44	4.08	0.048
	stand. amb.	oral	19.24	23.69	-4.45	n.s.
		vestibular	24.39	18.79	5.60	0.044
	stand. dark	oral	7.52	8.28	-0.75	n.s.
		vestibular	18.75	14.06	4.68	0.014
lower	clin.	oral	18.90	18.32	0.58	n.s.
		vestibular	23.03	28.99	-5.96	0.023
	stand. amb.	oral	17.98	16.20	1.78	n.s.
		vestibular	24.17	28.68	-4.51	n.s.
	stand. dark	oral	11.17	10.95	0.22	n.s.
		vestibular	21.46	27.43	-5.97	0.025

Teeth Mira						
jaws	setting	surface	MEAN canines	MEAN incisors	MEAN canines - MEAN incisors	p-value
upper	clin.	oral	19.22	27.33	-8.11	0.012
		vestibular	52.52	40.08	12.44	0.001
	stand. amb.	oral	16.64	25.73	-9.09	0.005
		vestibular	49.17	35.31	13.85	0
lower	clin.	oral	35.37	36.09	-0.72	n.s.
		vestibular	44.32	52.34	-8.02	0.023
	stand. amb.	oral	31.03	32.02	-0.99	n.s.
		vestibular	38.75	43.26	-4.51	n.s.

Surface QLF-D						
jaws	teeth	setting	MEAN vestibular	MEAN oral	MEAN vestibular - MEAN oral	p-value
upper	incisors	clin.	14.44	19.43	-4.98	n.s.
		stand. amb.	18.79	23.69	-4.90	n.s.
		stand. dark	14.06	8.28	5.79	n.s.
	canines	clin.	18.52	16.08	2.44	n.s.
		stand. amb.	24.39	19.24	5.15	n.s.
		stand. dark	18.75	7.52	11.22	0.001
lower	incisors	clin.	28.99	18.32	10.67	0.01
		stand. amb.	28.68	16.20	12.48	0.005
		stand. dark	27.43	10.95	16.47	0.001
	canines	clin.	23.03	18.90	4.13	n.s.
		stand. amb.	24.17	17.98	6.20	n.s.
		stand. dark	21.46	11.17	10.29	n.s.

Surface Mira						
jaws	teeth	setting	MEAN vestibular	MEAN oral	MEAN vestibular - MEAN oral	p-value
upper	incisors	clin.	40.08	27.33	12.75	n.s.
		stand. amb.	35.31	25.73	9.59	n.s.
	canines	clin.	52.52	19.22	33.30	0
		stand. amb.	49.17	16.64	32.52	0
lower	incisors	clin.	52.34	36.09	16.25	0.012
		stand. amb.	43.26	32.02	11.24	0.012
	canines	clin.	44.32	35.37	8.96	n.s.
		stand. amb.	38.75	31.03	7.72	n.s.

Supplementary table S1:

Table S1 Mean Plaque coverage (%) of QLF-D and conventional (disclosed plaque) images and method differences for different settings, jaws, tooth types and tooth surfaces. Abbreviations: clin. = clinical setting; stand. amb. = standardized setting, ambient light; stand. dark = standardized setting, dark; n.s. = not significant
